# A Visit to a Training School for the Deaf

**Published:** 1890-02-15

**Authors:** 


### A Visit to a Training School for the Deaf.

From a Travelling Correspondent.

Many who perhaps have unfortunately relations afflicted with deafness, may have felt keen discouragement after a visit to an Aural surgeon. There is no branch of medical or surgical practice beset with greater difficulties than this; and only too frequently the outlook for patients who have become victims of otitis and its consequences, or pharyngeal catarrh, with extension to the ear is depressing—at least, so far as the surgeon can come to their aid. But in another direction altogether one is happily astounded at the results which may be reached in these cases, and cases of congenital deafness. One has had occasion to observe that a deaf person often appears to understand through the eye, and to pick up the sense of what is spoken by observing the lines and movements of the speaker's mouth. By following up this clue many a deaf person has been trained so as to delude an observer into the belief that his ears must after all be efficient. A very gratifying visit to the Institution for the Blind and Deaf in the well-known university town of Zurich, in Switzerland, discovered the teacher instructing his junior class of some dozen boys and girls, whose ears were totally unable to hear. There was nothing peculiar in the physiognomy of the children, and their intelligence was evident from the sharp interest they took in the lesson; vieing with one another who should be first to answer the questions of their instructor. One young girl was remarkably clever, and did not labour under a thickness of utterance which was noticeable in the case of several of the other scholars. The age of the pupils in this elementary class varied from about seven to 12 years, and it appeared that instruction was not usually begun until the age of seven. The lesson was upon the birth of our Saviour in Bethlehem, how there was no vacant room in the inn, and how the shepherds watched their flocks by night. The teacher asked questions, not in a loud voice, but with much facial expression; suiting the action to the sense not only with gesture, but with movements of the arms and fingers, and changes of position, going, for instance, to the window as if the whole scene was being enacted outside. Then he kept time by pointing to the words written upon the blackboard. Thus taking a single sentence: "Joseph and Mary came to an inn, where they wished to pass the night," the teacher first of all deliberately and distinctly read the words, being followed by the children with close attention. But it was the movements of his mouth that they followed, correcting their impressions by what stood written on the blackboard. Then the teacher proceeded by way of question and answer :—

Q. Who came to an inn?

A. Mary and Joseph.

Q. Joseph and Mary came where?

A. To an inn.

Q. Why did Joseph and Mary come to an inn?

A. They wished to pass the night.

And so on.

If the first pupil questioned did not answer correctly, he was almost always taken down by the next. No single question puzzled the whole class. And how was it that these afflicted children, totally unable to hear, be it remembered, were yet intelligently following every question of the teacher? It was because they heard with their eyes, that is to say, they understood every movement of their teacher's mouth just as well as if they could hear the accompanying words, a result reached by long and patient teaching, with the writing on the blackboard to act as interpreter between teacher and taught. Hanging round the school-room were yet other interpreters in the shape of coloured pictures of birds, animals, and plants.

Passing into the next room, one found the senior pupils. There was a pair of globes on the table, and maps with more elaborate pictures than those we have just referred to on the walls. A youth of perhaps 16 was called forward, and asked to repeat after the teacher this long sentence: "This gentleman has come from England to visit our institution in Zurich and wishes to see what you can do." Without any hesitation, the lad repeated word for word the long sentence his teacher had just uttered without hitch or stumble. It was surprising. He could not hear with the ear one single syllable, but after assiduous and successful training he could hear through the eye every word spoken, by carefully observing the changing lines of his teacher's mouth. For every word the mouth had for him a form; its lines were a picture which he could read. That surprise on our part might not take refuge in a suspicion of sharp practice, we were ourselves invited to converse with the pupil. Then followed an examination *viva voce*, in our presence, upon the geography of England, in which the pupil was thoroughly well informed.

"But in the dark he would not be able to understand."

"No, he could not," said the teacher, with a gesture of discouragement; but making the youth shut his eyes, and taking the lad's hand in his own, he used it as a pen to write the word "table." "Table," said the pupil; but it is needless to multiply examples. In a word, during the day he would have passed himself off as in full possession of his faculty of hearing, so long as he could observe the face of his *vis-à-vis*.

Let any of your readers who are sceptical judge for themselves of the literal accuracy of these facts by a visit to some such institution, and let those patients whom our aural surgeons have dismissed, with however much pain and regret, saying, "I can do nothing for you," be assured that there is yet a chance for them. There is no reason why they should not, by assiduous training, become very skilful in hearing by sight, and pass themselves off in most companies, not as individuals wanting one precious sense, but as in the ample possession of the power to hear and understand.

